# Measurement of Colonic Transit Time Based on Radio Opaque Markers in Patients with Chronic Idiopathic Constipation; A Cross-Sectional Study

**DOI:** 10.5812/ircmj.16617

**Published:** 2013-12-05

**Authors:** Hazhir Saberi, Nastaran Asefi, Amir Keshvari, Shahram Agah, Mohsen Arabi, Hoda Asefi

**Affiliations:** 1 Department of Radiology, Tehran University of Medical Sciences, Tehran, IR Iran; 2 Department of Surgery, Tehran University of Medical Sciences, Tehran, IR Iran; 3 Department of Gastroenterology, Rasool Akram Hospital, Tehran, IR Iran; 4 Department of Internal Medicine, Rasool Akram Hospital, Tehran, IR Iran

**Keywords:** Constipation, Habits, Hypothyroidism

## Abstract

**Background:**

Constipation is one of the most common gastrointestinal disorders particularly in industrialized countries. Incidence of constipation varies from 3.4 % to 27.2% in different societies. Increase in urban population, industrialization of communities, changes in behavioral and nutritional habits and inactivity have increased the number of patients suffering from constipation.

**Objectives:**

The aim of the study was to measure colonic transit time in patients with chronic idiopathic constipation.

**Patients and Methods:**

Fifty-two patients with chronic idiopathic constipation (according to ROME III criteria) were selected. Patients with diabetes mellitus, hypothyroidism, and hypoparathyroidism were excluded. Each patient took a capsule containing ten 1-3 mm long angiographic guide daily for 6 days. Abdominal x-ray was performed on the seventh day. Remaining markers in each segment were counted and segmental and total colonic transit time was calculated. The analysis was performed by SPSS version 18. In all tests, a P-value less than 0.05 was considered statistically significant.

**Results:**

The mean age of patients was 36.9 ± 10.1 years. 80.8 % of patients were female. The mean total colonic transit time was 40.8 ±35.1 hours. 34.6 % of patients and 36.5 % of them had prolonged total and segmental colonic transit time.

**Conclusions:**

We found that prolonged total and segmental colonic transit time is common in patients with chronic idiopathic constipation.

## 1. Background

Constipation is one of the most common gastrointestinal disorders particularly in industrial countries (1, 2). Incidence of constipation varies from 3.4 % to 27.2% in different societies (3). Increase in urban population, industrialization of communities, changes in behavioral and nutritional habits, and inactivity have increased the number of patients suffering from constipation (4). Incidence of constipation increases by aging, especially after 65 years of age (3, 5). In this age group 26% of males and 34% of females have constipation (6-8). The general incidence of constipation is twice in women. It is also more common in urban populations, lower socioeconomic classes, and in people with lower level of education (3, 9). Constipation has different definitions. It can be described as defecation less than three times a week; however, this definition is not widely accepted (10). International functional committee has suggested ROME III diagnostic criteria for the diagnosis of functional constipation (11, 12). Constipation has different etiologies and it can be the first clinical sign of metabolic disorders such as diabetes mellitus, hypoparathyroisism, hypercalcemia, neurologic disorders, obstructive bowel disorders and sometimes medications side effects (13, 14). Constipation would not be categorized as functional unless no specific etiology is determined. Chronic idiopathic constipation is usually seen in women. Symptoms would not be relieved by adding more fiber to the diet (15, 16).

Idiopathic constipation is divided into three subtypes: 1) Constipation with normal colonic transit, which is often associates with psychosocial abnormalities (17, 18); 2) Colonic inertia (slow transit constipation). Most of the patients with severe constipation and abnormal colonic transit have colonic inertia, that in this case, transit of particles via colon is slow; 3) Outlet obstruction, in which food particles pass normally through the colon but slowdown in rectum (19, 20). The first step in the evaluation of constipation is obtaining a precise history of underlying medical disorders such as diabetes mellitus, hypoparathyroidism, hypothyroidism, and hypercalcemia and if needed, measuring the serum levels of TSH, T4, AlkP, P, Ca, FBS, and CBC and stool exam.

Other diagnostic evaluations include colonoscopy, barium enema, and evaluation of colonic transit time (CTT) by marker and anorectal manometer (21-23). Evaluation of CTT is a useful method for chronic idiopathic constipation. In this method, the patient takes a specific number of radio opaque markers and abdominal X-ray is performed at the determined time (24, 25). Performing CTT makes is possible to specify which colonic segment has abnormal motility and is the cause of constipation (26). It is also useful in distinguishing patients with Irritable bowel syndrome (IBS) that may benefit from subtotal colectomy and ileorectostomy (27, 28).

## 2. Objectives

We aimed to distinguish subtypes of chronic idiopathic constipation by evaluating the colonic transit time, so that the appropriate treatment would be assigned to each patient.

## 3. Patients and Methods

In this cross-sectional study, we evaluated patients with constipation that were referred to the gastrointestinal and surgical clinics of Imam Khomeini hospital complex, Tehran, Iran. Patients with the history of known diabetes mellitus, hyperparathyroidism, hypoparathyroidism, and hypothyroidism were excluded. Patients were included according to ROME III criteria (Table 1)

**Table 1.** Diagnostic Criteria for Functional Constipation

Presence of Defecation Discomfort During at Least 3 Months in the Past 6 Months
**1) Two or more of the following symptoms**
a) Straining during at least 25 % of defecation
b) Lumpy or hard stools in at least 25 % of defecation
c) Sensation of incomplete evacuation for at least 25 % of defecation
d) Sensation of anorectal obstruction or blockage for at least 25 % of defecation
e) Manual maneuvers to facilitate at least 25% of defecation (e.g. digital evacuation, support of pelvic floor)
f) Fewer than three episodes of defecation per week
**2) Loose stools are rarely present without use of laxatives**
**3) Insufficient criteria for irritable bowel syndrome: (recurrent abdominal pain or discomfort at least 3 days/month in the last 3 months associated with two or more of the following**
a) Improvement with defecation
b) Onset associated with a change in the frequency of defecation
c) Onset associated with a change in the stool appearance)

Although CTT could be performed in different ways, we chose the Arhan method because of single X-ray exposure and no need for repeated referal to the radiology department. In this method, the patients were given capsules containing ten 1-3 millimeter-long marker made of angiographic catheter. Patients should not have used laxatives during the week before CTT study. They took a capsule at 9 am for six consecutive days and came for taking abdominal X-ray in the 7th day morning.

**Figure 1.**
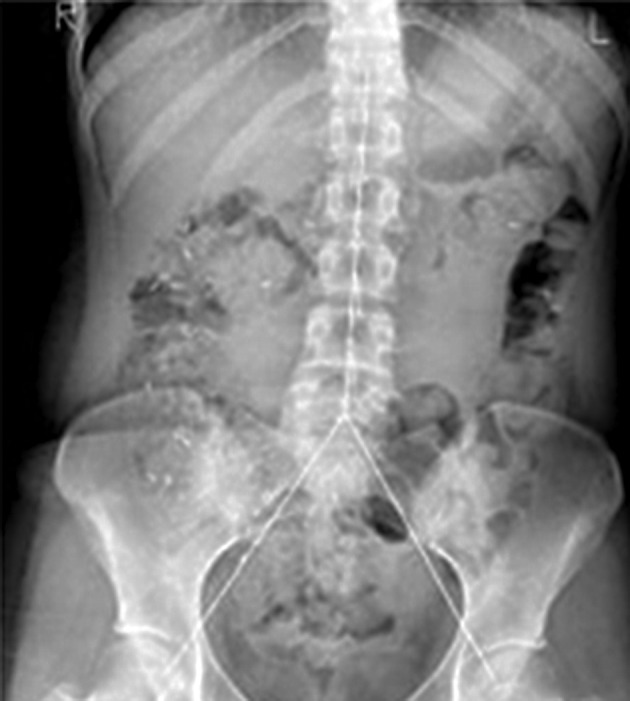
The plain Abdominal x-ray is Divided into 3 Segments and Radiopaque Markers are Counted in Each Segment

All the plain abdominal X-rays were evaluated by a single radiologistand the number of markers in each segment (right, left and rectosigmoid) was counted as shown in Figure 1. 

Total, right, left and rectosigmoid colonic transit time was computed using this formula:

CTT= 2.4×N

N= number of available markers in each segment or totally

The data was then analyzed using the statistical software SPSS 18.0.0. (SPSS Inc. Chicago, IL, USA). The t test was used to compare two groups and P-value lower than 0.05 was considered significant.

## 4. Results

Fifty-two patients fulfilled the inclusion criteria. The mean age of patients was 36.9 ± 10.1 years. (Range: 18-66 years). Forty-two patients (80.8 %) were female. We also found that 28 patients (53.8%) had history of chronic constipation for more than 3 years. In this study, the mean total colonic transit time was 40.8±35.1 hours while in rectosigmoid, ascending colon and descending colon, it was 15.5 ± 12.4 , 14.7 ± 10.8 and 12.9 ± 9.5 hours, respectively (Table 1). In 18 patients (34.6%), total colonic transit time was longer than normal (≥45 hours) (Table 2). 

**Table 2.** Frequency of Normal and Abnormal Colonic Transit Time

Total Colonic Transit Time	Number	Percent
**Normal (<45 hr)**	34	65.4
**Abnormal (>45 hr)**	18	34.6
**Total **	52	100

From 34 patients with normal total colonic transit time (13), 19 patients (36.5 %) had abnormal segmental transit time (≥ 15 hours). among these patients, 10 had prolonged right colonic transit time, while 6 and 3 patients had prolonged rectosigmoid and left colonic transit time, respectively.

The mean total colonic transit time was 42 ± 35.4 and 35.2 ± 32.1 hours in women and men, respectively, with no statistically significant difference between them (P = 0.57). The mean total colonic transit time was highest in patients older than 50 years and was lowest in patients less than 30 years old (Table 3). 

**Table 3.** Mean Colonic Transit Time in Different Age Groups

Age, (y)	Number	Mean ± SD (h)
**≤ 30**	12	38.6 ± 36.2
**30 – 40**	22	39.1 ± 36.7
**40 – 50**	13	44.2 ± 40.4
**≥ 50**	5	49.4 ± 42.1

Regarding the mean total colonic transit time, no significant difference was found among different age groups and pair comparing (P > 0.05). there was no statistically significant difference with regard to the mean total colonic transit time between patients with less or equal to three years history of constipation (38.6 ± 17.08 hours) and those with more than three years history of constipation (P = 0.67).

## 5. Discussion

To put in a nutshell, the aim of this study was to evaluate colonic transit time in patients with chronic idiopathic constipation.

In our study, among 52 patients who entered the study, 18 (34.6%) had prolonged total colonic transit time. In similar studies, the frequency was different in patients with idiopathic constipation (29). In a study published by Lopes et al. (30) in 2008, of 30 patients with constipation, eight patients had prolonged total colonic transit time, which was similar to our study findings. in contrast, in a study by Mollen et al. (31) of 112 patients with constipation, 70% had prolonged total colonic transit time, which was higher than the rate of this condition in our patients (34.6%). In contrary to our study, they obtained the x-ray images after 10 days of administrating radio opaque markers, while we took the images on the seventh day. In our study, 36.5% of patients with normal total colonic transit time had prolonged segmental transit time. In 10 patients’ right colon was affected while in six and three patients, rectosigmoid and left colon were affected, respectively.

Lopes et al. (30) found that nine, eight, and three out of 22 patients with normal total colonic transit time, had isolated slow transit in right colon, rectosigmoid, and left colon, respectively. The frequency of prolonged segmental colonic transit time was higher in right colon which was similar to our findings. The mean segmental colonic transit time was longer in rectosigmoid in our study. In a similar study by Zaslavsky et al. (32) on 13 patients with constipation, the mean total colonic transit time was 58.6 ± 17.4 hours while segmental colonic transit time in rectosigmoid, right, and left colon were 17.2 ± 16.2 , 15.9 ± 12.4, and 14.7 ± 13.4, respectively. 

With regard to longer mean transit time in right colon, subtotal surgery could be more helpful in the case of resistance to medical treatment. The mean total colonic transit time was higher in women in our study but this was not statistically significant (P ≥ 0.05). Danquechin et al. (33), measured colonic transit time in 82 healthy people, and found that total colonic transit time in women was longer than men.

In conclusion, abnormal total and segmental colonic transit time is common in patients with chronic idiopathic constipation; hence, in the case of inappropriate response to medical treatment, subtotal colectomy would be helpful.
